# The increasing importance of *Plasmodium ovale* and *Plasmodium malariae* in a malaria elimination setting: an observational study of imported cases in Jiangsu Province, China, 2011–2014

**DOI:** 10.1186/s12936-016-1504-2

**Published:** 2016-09-07

**Authors:** Yuanyuan Cao, Weiming Wang, Yaobao Liu, Chris Cotter, Huayun Zhou, Guoding Zhu, Jianxia Tang, Feng Tang, Feng Lu, Sui Xu, Yaping Gu, Chao Zhang, Julin Li, Jun Cao

**Affiliations:** 1 Key Laboratory of National Health and Family Planning Commission on Parasitic Disease Control and Prevention, Jiangsu Provincial Key Laboratory on Parasite and Vector Control Technology, Jiangsu Institute of Parasitic Diseases, Wuxi, 214064 Jiangsu People’s Republic of China; 2 Malaria Elimination Initiative, Global Health Group, University of California, San Francisco (UCSF), San Francisco, CA USA; 3 Public Health Research Center, Jiangnan University, Wuxi, 214122 Jiangsu People’s Republic of China

**Keywords:** *Plasmodium ovale*, *Plasmodium malariae*, Importation, Misdiagnosis, Latency period, GIS, Malaria elimination

## Abstract

**Background:**

Following initiation of China’s National Malaria Elimination Action Plan in 2010, indigenous malaria infections in Jiangsu Province decreased significantly. Meanwhile imported *Plasmodium* infections have increased substantially, particularly *Plasmodium ovale* and *Plasmodium malariae*. Given the risk for malaria resurgence, there is an urgent need to understand the increase in imported *P. ovale* and *P. malariae* infections as China works to achieve national malaria elimination.

**Methods:**

An observational study of imported malaria cases in Jiangsu Province, China was carried out for the period of 2011–2014.

**Results:**

A total of 1268 malaria cases were reported in Jiangsu Province from 2011 to 2014. Although imported *Plasmodium falciparum* cases (n = 1058) accounted for 83.4 % of all reported cases in Jiangsu, *P. ovale* cases (14, 19, 30, and 46) and their proportion (3.7, 9.6, 8.8, and 13.0 %) of all malaria cases increased over the 4 years. Similarly, *P. malariae* cases (seven, two, nine, and 10) and proportion (1.9, 1.0, 2.6, and 2.8 %) of all malaria cases increased slightly during this time. A total of 98 cases of *Plasmodium ovale curtisi* (47/98, 48 %) and *Plasmodium ovale wallikeri* (51/98, 52 %) were identified as well. Latency periods were significant among these *Plasmodium* infections (p = 0.00). Also, this study found that the latency periods of *P. ovale* sp.*, P. malariae* and *Plasmodium vivax* were significantly longer than *P. falciparum*. However, for both *P. ovale curtisi* and *P. ovale wallikeri* infections, the latency period analysis was not significant (p = 0.81). Misdiagnosis of both *P. ovale* and *P. malariae* was greater than 71.5 and 71.4 %, respectively. The *P. ovale* cases were misdiagnosed as *P. falciparum* (35 cases, 32.1 %), *P. vivax* (43 cases, 39.4 %) by lower levels of CDCs or hospitals. And, the *P. malariae* cases were misdiagnosed as *P. falciparum* (ten cases, 35.7 %), *P. vivax* (nine cases, 32.1 %) and *P. ovale* sp. (one case, 3.6 %). Geographic distribution of imported *P. ovale* sp. and *P. malariae* cases in Jiangsu Province mainly originated from sub-Saharan Africa such as Equatorial Guinea, Nigeria, and Angola.

**Conclusions:**

Although the vast majority of imported malaria cases were due to *P. falciparum*, the increase in other rare *Plasmodium* species originating from sub-Saharan Africa and Southeast Asia should be closely monitored at all levels of health providers focusing on diagnosis and treatment of malaria. In addition to a receptive vector environment, long latency periods and misdiagnosis of *P. malariae* and *P. ovale* sp. increase the risk of re-introduction of malaria in China.

## Background

Malaria is one of the most important public health problems worldwide. Globally, an estimated 3.3 billion people in 97 countries and territories are at risk of malaria. An estimated 214 million malaria cases occurred globally in 2015 and led to 438,000 deaths [1]. Malaria was once a major challenge in China, however after large-scale implementation of interventions such as mass drug administration [2], indoor residual spraying and long-lasting insecticide nets, China has effectively controlled malaria and reduced local malaria to 56 cases in 2014 [3].

After China began implementing the national malaria elimination action plan (NMEAP) [4] in 2010, the ‘1-3-7’ approach, which defined targets to guide and monitor case reporting, investigation and response, respectively, was carried out and local malaria infections across the country and in Jiangsu Province decreased significantly [5]. However, imported *Plasmodium falciparum* malaria cases, mostly from overseas migrant workers posed a challenge [6].

For *Plasmodium ovale* sp. and *Plasmodium malariae*, only sporadic malaria cases have been reported in China [7–11]. Historically, these two species of *Plasmodium* are rare in China. Since 1960, there have been no reported local *P. malariae* cases in Jiangsu Province. Given their rarity, local centres for disease control and prevention (CDC) and hospital microscopy examiners commonly misdiagnose *P. ovale* and *P. malariae* as *Plasmodium vivax* and *P. falciparum,* respectively [12, 13]. Due to the similarity in the parasite density and life cycle forms of ovale malaria and vivax malaria, *P. ovale* sp. is easily and frequently misdiagnosed as *P. vivax* [14, 15]. If misdiagnosed as *P. falciparum, P. ovale* sp. malaria cases could relapse because drug treatments between the two species differ. Furthermore, treatment based on an incorrect diagnosis of *Plasmodium* species leads to misuse of anti-malarial drugs as well as drug wastage.

With the substantially increasing number of imported *P. ovale* and *P. malariae* cases, it is necessary to understand the difference between these species. At present, there are few epidemiological studies focused on imported *P. ovale* and *P. malariae* cases in China. In this work, by collecting and analysing data from the diseases surveillance information system for the period 2011–2014, this study describes *P. ovale* sp. and *P. malariae* prevalence trends, population characteristics, latency periods, and geographic distribution patterns to provide a basis for the prevention and control of imported *P. ovale* sp. and *P. malariae* malaria in Jiangsu Province.

## Methods

### Study site

**Fig. 1:**
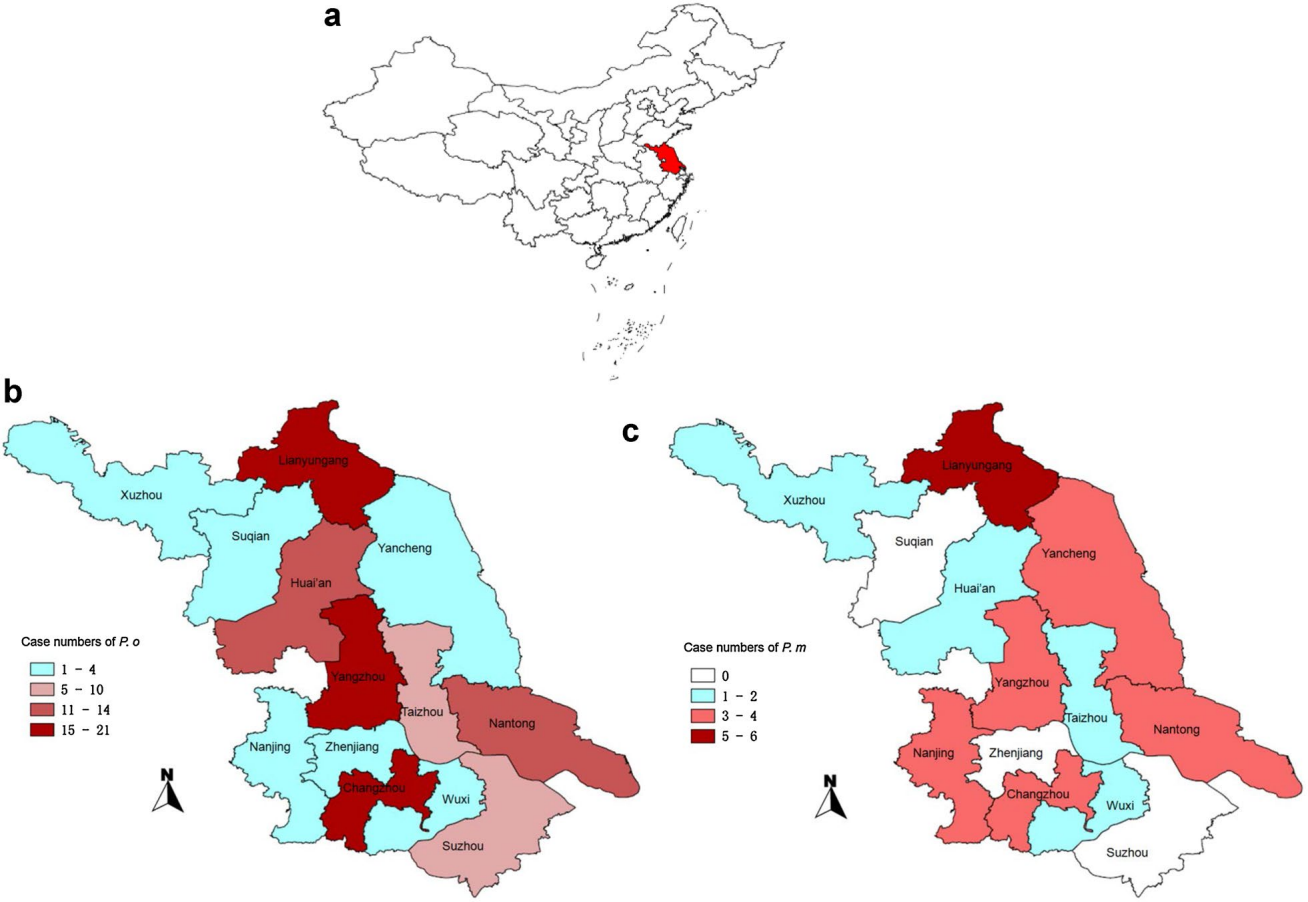
Geographic distribution of imported malaria cases in Jiangsu Province. **a** Location of Jiangsu, China; **b** *P. ovale* sp.; **c** *P. malariae*

Jiangsu Province, located in the southeast of China (Fig. 1a) has sub-tropical and warm temperate zones and an annual average temperature of 14.7 °C (range of 3.0–25.9 °C) [16]. Annual rainfall in Jiangsu is 1000.4 mm, mainly concentrated from June to September (malaria transmission season). The climate and environment is suitable for the breeding of *Anopheles*, including *Anopheles sinensis* and *Anopheles anthropophagus* [17, 18].

### Data extraction

China’s routine diseases surveillance information system (CRDSIS) maintains an electronic database of malaria cases; it was established by China Centres for Disease Control and Prevention (China CDC) in 2004. Data on all *Plasmodium* species in Jiangsu Province from 2011 to 2014 were downloaded from the CRDSIS database. Reporting districts include 13 prefecture CDCs and more than 100 county CDCs across Jiangsu. All malaria cases, including gender, age, occupation, admission dates, symptom onset time, and time of diagnosis were reported from prefectures and county CDCs in Jiangsu. Detailed demographic and clinical data, as well as travel history, were obtained from follow-up case investigation reports.

### Species confirmation

Parasite species of all malaria cases were initially identified by examination of Giemsa-stained thick and thin smears under oil immersion at 500–1000× magnification microscopy by microscopists at basic levels of CDCs and hospitals. Through the malaria diagnosis reference laboratory in Jiangsu Institute of Parasitic Diseases (JIPD), species designations of cases were confirmed using PCR. Parasite species were distinguished by PCR amplification, using methods published [19]. Real-time PCR assay for discrimination of *Plasmodium ovale curtisi* and *Plasmodium ovale wallikeri* was carried out by real-time PCR assay method [20]. PCR reagents used were from Shanghai Sangon Biotech Corp.

### Data analysis

The datasets established included basic characteristics of all cases and input into Excel (Microsoft Office 2007). Characteristics were stratified and analysed by gender, age group and occupation. Categorical variables were carried out by Chi square tests with Fisher’s exact correction when the expected frequency in any cell was five or less. The time elapsing, in days, was calculated for each episode of malaria cases by subtracting arrival dates when patients (migrant workers overseas) arrived back to China from the dates of symptom onset for all malaria cases. Arrival dates were the last possible point in time when parasites could have been introduced by an infectious *Anophele*s bite. The days elapsing approximated with latency periods. Days elapsing between arrival and symptom onset for all malaria cases were distributed by box-plots. Because latency periods have a non-normal distribution, and one-way ANOVA tests were conducted after rank transformation. Two-tailed *t*-tests were used and *p*-*values* less than 0.05 were considered statistically significant. All statistical analyses were performed in STATA v.12 (Timberlake, College Station, TX, USA) and SPSS v.16.0 (Statistical Product and Service Solutions). Geographic information system (GIS)-based spatial analysis was conducted to identify geographic distribution patterns of malaria cases at high risk at the provincial and national levels. All the spatial analysis was carried out by QGIS (Quantum GIS) v2.10.1.

## Results

### Epidemiological profiles of *Plasmodium ovale* and *Plasmodium malariae*

From 2011 to 2014, a total of 1268 malaria cases were reported in Jiangsu Province. Although imported *P*. *falciparum* cases (n = 1058) accounted for 83.4 % of all reported cases, *P. ovale* sp. cases (14, 19, 30, 46) and proportion (3.7, 9.6, 8.8, 13.0 %) of total malaria cases increased over the 4 years. Similarly, *P. malariae* cases (7, 2, 9, 10) and proportion (1.9, 1.0, 2.6, and 2.8 %) of total malaria cases increased slightly during this time period. Mixed infection cases include six *P. ovale* sp. cases co-infected with *P. falciparum* and two *P. malariae* cases co-infected with *P. falciparum* (Table 1).

**Table 1:** Imported malaria cases in Jiangsu Province, 2011–2014

Year	All cases	*P. falciparum*	*P. vivax*	*P. ovale* sp.	*P. malariae*	Mixed infection
		N (%)	N (%)	N (%)	N (%)	N (%)
2011	374	306 (81.8)	47 (12.6)^a^	14 (3.7)	7 (1.9)	0 (0)
2012	198	171 (86.4)	6 (3.0)	19 (9.6)	2 (1.0)	0 (0)
2013	341	289 (84.8)	8 (2.3)	30 (8.8)	9 (2.6)	5 (1.5)
2014	355	292 (82.3)	4 (1.1)	46 (13.0)	10 (2.8)	3 (0.8)
Total	1268	1058 (83.4)	65 (5.1)	109 (8.6)	28 (2.2)	8 (0.7)

^a^ Including 13 indigenous vivax malaria in 47

After the NMEAP launched in 2010, only 13 indigenous vivax malaria cases out of 47 total vivax malaria cases were recorded in Jiangsu in 2011. From 2012, local vivax malaria cases in Jiangsu sharply decreased to zero and all *P. vivax* cases were imported from abroad. In contrast to *P. malariae* and *P. ovale* sp., the vivax malaria number and its proportion in all cases decreased during the time period (Table 1).

### Demographic characteristics of imported ovale and malariae malaria

Of the 145 ovale and malariae malaria cases which including mixed infection (Table 2), nearly all were male (139/145, 95.9 %). All the cases were ages from 20 to 60 years and in the 41–50 years age group, *P. ovale* sp. and *P. malariae* accounted for 58.3 and 56.7 %, respectively. For occupation, most cases occurred in migrant workers [*P. ovale* (113, 98.3 %); *P. malariae* (28, 93.4 %)]. Also, there was one foreigner identified as a *P. ovale* and *P. malariae* case, respectively.

**Table 2:** Demographic characteristics of imported *Plasmodium malariae* and *Plasmodium ovale*, 2011–2014

Variables	*P. ovale* sp. (n = 115)	*P. malariae* (n = 30)
	N (%)	N (%)
Gender		
Male	112 (97.4)	27 (90)
Female	3 (2.6)	3 (10)
Age groups		
≤20	0 (0)	0 (0)
21−30	16 (13.9)	6 (20)
31−40	25 (21.7)	6 (20)
41−50	67 (58.3)	17 (56.7)
≥51	7 (6.1)	1 (3.3)
Occupation		
Migrant workers	113 (98.3)	28 (93.4)
Students	1 (0.9)	1 (3.3)
Foreigners	1 (0.9)	1 (3.3)

### Latency analysis

**Fig. 2:**
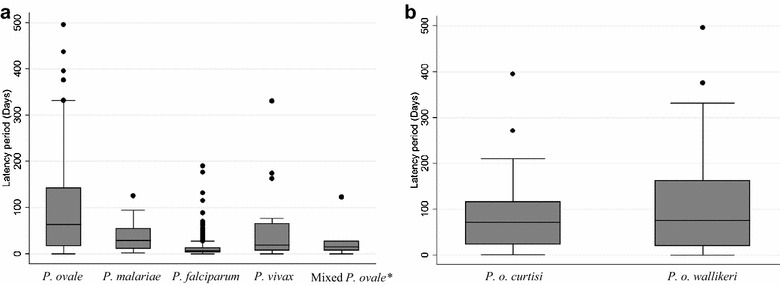
Latency periods for malaria cases in Jiangsu Province. **a** All cases; **b** *P. ovale curtisi* and *P. ovale wallikeri* cases. The time elapsing, in days, for each episode of malaria was calculated by subtracting arrival date when patient (migrant worker overseas) returned back to China from onset of symptom date. The *midline* of each box-plot is the median, with the edges of the box representing the interquartile intervals. Whiskers delineate the 5th and 95th percentiles. The *black dots* mean the outliers of days elapsing between arrivals in China and onset and these outliers were removed when statistical analysis was conducted. The mixed *P. ovale* cases were six *P. ovale* sp. cases co-infected with *P. f.* Because there were only two *P. malariae* cases co-infected with *P. f* and the number of mixed *P. malariae* cases didn’t satisfy with box-plot

The median number of days elapsing (latency periods) between individuals arriving in China and the time of symptom onset of malaria cases were given for all patients. The median and interquartile range of *P. ovale* latency period was 63.5 days (17–143). The median and interquartile range of *P. malariae* latency period was 29.5 days (11–55.5). Conversely, the median and interquartile range of *P. falciparum* and *P. vivax* latency periods were only 7 days (3–13) and 19 days (8–63.5), respectively. The median and interquartile range of mixed *P. ovale* latency period was 15.5 days (8–28). Because there were only two *P. malariae* cases co-infected with *P. falciparum* and the number of mixed *P. malariae* cases didn’t satisfy with box-plot (Fig. 2a).

Through rank transformation and ANOVA for the latency periods of all malaria infections, the Bartlett test for equal variances is homogeneous (*χ*^*2*^ = 2.39*, p* = 0.66) and found that latency periods were significant among these *Plasmodium* infections (*F* = 52.34*, p* = 0.00). After using the Scheffe multiple comparisons test for latency periods of the four *Plasmodium* species, it found that the latency periods of *P. ovale, P. malariae* and *P. vivax* were significantly longer than *P. falciparum (p* = 0.000, 0.000 and 0.02*),* respectively. For latency period analysis of both *P. ovale curtisi* and *P. ovale wallikeri* infections, their median and interquartile range of latency periods were 71.5 (23.5–116) and 75 (20–162), respectively (Fig. 2b). However, this study showed the latency periods are not different between *P. ovale curtisi* and *P. ovale wallikeri* for latency periods *(t* = −0.24*, p* = 0.81*)*.

### Misdiagnosis of ovale and malariae malaria cases

Initial malaria diagnosis is carried out by microscopy at the laboratories of the lower level CDCs or hospitals (township or county), and confirmation was done by PCR at the provincial reference laboratory in JIPD. For the real prevalence, we separated the mixed infection cases which listed in Table 1. As shown in Table 3, of the 109 *P. ovale* malaria cases, only 31 cases had an initial diagnosis that was correct, accounting for 28.4 % of total *P. ovale* cases. The remaining *P. ovale* cases were misdiagnosed as *P. falciparum* (35 cases, 32.1 %), *P. vivax* (43 cases, 39.4 %) by lower levels of CDCs or hospitals. Similarly, of the 28 *P. malariae* cases only eight cases were initially diagnosed correctly accounting for 28.6 %. The remaining *P. malariae* cases were misdiagnosed as *P. falciparum* (ten cases, 35.7 %), *P. vivax* (nine cases, 32.1 %) and *P. ovale* sp. (one case, 3.6 %). The misdiagnosis of both *P. ovale* sp. and *P. malariae* was more than 71.5 and 71.4 %, respectively. In total, 1054 cases were initially diagnosed correctly, accounting for 99.6 % of the total *P. falciparum* cases. Of the 65 vivax malaria cases, 60 were initially diagnosed correctly, accounting for 92.3 %. Significant differences were observed among confirmed diagnosis cases and initially diagnosed cases (*χ*^*2*^ = 768.9, p = 0.000).

**Table 3:** Misdiagnosis when all malaria cases diagnosed firstly at lower levels of CDCs and hospitals in Jiangsu

Initial diagnosis	Confirmed diagnosis by PCR				Total
	*P. ovale* sp.	*P. malariae*	*P. falciparum*	*P. vivax*	
*P. ovale*	31	1	0	0	32
*P. malariae*	0	8	1	0	9
*P. falciparum*	35	10	1054	5	1104
*P. vivax*	43	9	3	60	115
Total	109	28	1058	65	1260

Pearson’s χ^2^ = 768.9 , *p* = 0.000

### Geographic distribution of *Plasmodium ovale* and *Plasmodium malariae* reporting

All 13 prefectures of Jiangsu Province reported ovale malaria cases imported from other countries, with the cases concentrated in Changzhou (21 cases, 18.3 %), Yangzhou (15, 13.0 %), Lianyungang (15, 13.0 %), and Nantong (13, 11.3 %) (Fig. 1b). However, ten prefectures reported *P. malariae* cases imported from other countries and cases were concentrated in Lianyungang (six, 20.0 %) (Fig. 1c).

### Geographic distribution of *Plasmodium ovale* and *Plasmodium malariae* originating in sub-Saharan Africa

**Fig. 3:**
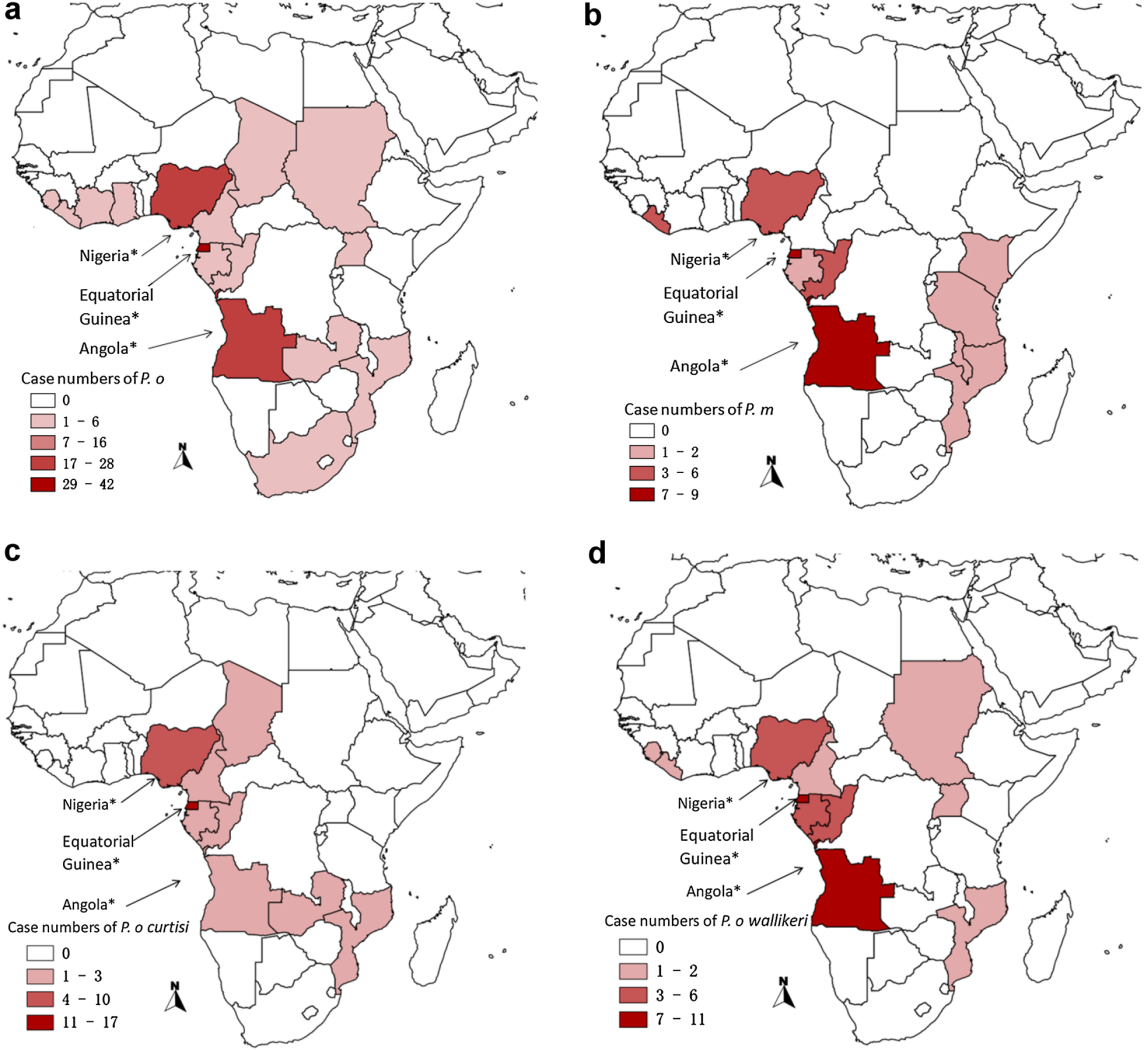
Geographic distribution of *Plasmodium ovale, Plasmodium malariae* and sub-species of *Plasmodium ovale curtisi and Plasmodium ovale wallikeri* cases originating from sub-Saharan Africa, respectively. **a** *P. ovale* sp.; **b** *P. malariae*; **c** *P. ovale curtisi*; **d** *P. ovale wallikeri*. *Asterisk* labels listed for only top 2–3 countries. There were only three *P. ovale* sp. cases imported from two Southeast Asian countries (Pakistan and Brunei) and only one *P. malariae* case imported from Pakistan

Nearly all ovale malaria cases were from sub-Saharan Africa (112/115, 97.4 %), with the top three countries from where ovale malaria was imported being Equatorial Guinea (41, 35.7 %), Nigeria (27, 23.5 %), and Angola (17, 14.8 %), accounting for 74 % of imported *P. ovale* sp. cases (Table 4; Fig. 3a). Three *P. ovale* cases from two Southeast Asian countries (Pakistan and Brunei) were also imported. Nearly all *P. malariae* cases were imported from sub-Saharan Africa (29/30, 96.7 %) as well, with the top three countries being Equatorial Guinea (9, 30.0 %), Angola (7, 23.3 %), and Nigeria (3, 10 %) accounting for 63.3 % of *P. malariae* cases (Fig. 3b). There was only one *P. malariae* case from a Southeast Asian country (Pakistan).

**Table 4:** Origin of imported *P. ovale* sp. and *P. malariae*

Country	*P. ovale* sp.		*P. malariae*		Total
	N	%	N	%	
Angola	17	14.8	7	23.3	24
Brunei	1	0.9	0	0	1
Cameroon	2	1.7	0	0	2
Chad	1	0.9	0	0	1
Cote d’Ivoire	1	0.9	0	0	1
Equatorial Guinea	41	35.7	9	30	50
Gabon	5	4.3	1	3.3	6
Ghana	2	1.7	0	0	2
Kenya	0	0	1	3.3	1
Liberia	1	0.9	3	10	4
Malawi	0	0	1	3.3	1
Mozambique	3	2.6	1	3.3	4
Nigeria	27	23.5	3	10	30
Pakistan	2	1.7	1	3.3	3
Sierra Leone	2	1.7	0	0	2
South Africa	1	0.9	0	0	1
Sudan	1	0.9	0	0	1
Tanzania	0	0	1	3.3	1
The Republic of Congo	6	5.1	2	6.9	8
Uganda	1	0.9	0	0	1
Zambia	1	0.9	0	0	1
Total	115	100	30	100	145

Totally 115 ovale cases were confirmed, but only 98 cases was identified as *curtisi* (47/98, 48 %) or *wallikeri* (51/98, 52 %), other 17 samples are confirmed as *P. ovale* sp., but not able to be identified.

### Origin of *Plasmodium ovale curtisi* and *Plasmodium ovale wallikeri* in sub-Saharan Africa

*Plasmodium ovale curtisi* species was concentrated to Equatorial Guinea (24 cases, 51.0 %), Nigeria (13 cases, 27.7 %), and Angola (3 cases, 8.5 %) (Fig. 3c). However, *P. ovale wallikeri* was mainly distributed in Angola (14 cases, 27.5 %), Equatorial Guinea (13 cases, 25.5 %), and Nigeria (8 cases, 15.7 %) (Fig. 3d).

## Discussion

These results show long latency periods and frequent misdiagnosis by microscopy of malariae and ovale malaria cases and highlight a challenge that all levels of CDCs should focus on for accurate diagnosis and surveillance of rare *Plasmodium* species imported from sub-Saharan Africa and Southeast Asia. The local malaria situation in China has been effectively controlled, however, as local malaria cases have decreased drastically, imported malaria cases are now the main challenge for China to reach elimination. To address this challenge of misdiagnosing malaria infections, the Chinese Government has held training at different levels, including a national competition for the diagnosis of parasitic diseases [21]. The training and competitions have helped to maintain the microscopy capabilities for accurate detection and diagnosis of *Plasmodium* parasites among professionals at all levels of CDCs and hospitals.

In recent years, the total number of imported malaria cases coming from abroad to Jiangsu Province ranked in the top three provinces in China [22, 23]. Although imported falciparum malaria cases accounted for a majority of the total cases in this study, the other three *Plasmodium* species (*P. ovale* sp.*, P. malariae,* and *P. vivax*) were detected every year during the 2011–2014 period. Ovale and malariae malaria cases increased overall during the study period, increasing the potential risk of re-introducing malaria in Jiangsu Province, an area with historically stable malaria transmission [18]. Vivax and falciparum malaria outbreaks have been reported in non-endemic areas in 2005 and 2007, respectively [24, 25] and pose a major challenge for Jiangsu Province to maintain zero incidence of locally transmitted malaria. Given that the transmitting vectors *A. sinensis* and *A. anthropophagus* are present, as well as having a suitable climate [17, 18], the potential for local transmission in Jiangsu remains.

Symptoms such as fever, sweating and headache develop on average 7 days after migrant workers return to Jiangsu Province. Of those with *P. ovale* sp. and *P. malariae* infections, the average number of days before fever onset was almost 2 months and nearly 1 month, respectively. During these long periods some *Plasmodium*-infected individuals could have asymptomatic parasitaemia, providing an opportunity for mosquitoes to bite and further contribute to local transmission. Additionally, these asymptomatic individuals may donate their blood through local blood stations without knowing they are infected with malaria. A high rate of antibodies against *P. ovale* and *P. malariae* were found in asymptomatic blood donors in Western Africa [26].

Due to the rare occurrence of indigenous ovale and malariae malaria cases in China, these *Plasmodium* species have been largely neglected. Nevertheless, severe cases of *P. ovale* sp. pertaining to acute respiratory symptoms and renal failure have been reported [27–29]. *Plasmodium ovale* cases have the potential to relapse if misdiagnosed as *P. falciparum* and incorrect treatment provided. Furthermore, treatment based on an incorrect diagnosis leads to misuse of anti-malarial drugs as well as drug wastage. Also, *P. ovale* infections have been misidentified in clinical laboratories settings as *P. vivax* among malaria cases imported to Singapore over the last 3 years. Misidentified *P. ovale* infections are reported for the first time among imported malaria cases in Singapore [15]. Although both *P. ovale* sp. and *P. malariae* cause mild symptoms, *P. malariae* can cause chronic nephritic syndrome, leading to adverse reactions during treatment and a high rate of mortality [30]. In Hunan Province, China, *P. malariae* infections can cause sudden attacks in the following years when patients go without proper anti-malarial treatment [7]. As countries reduce their malaria burden, strategies that address the changing epidemiology to increasing proportions of infections from non-falciparum species need to be developed, validated and adopted [31]. Ensuring a high capacity potential for the diagnosis of falciparum and vivax malaria, as well as for *P. ovale* sp. and *P. malariae* infections, remains a challenge to all levels of CDCs and hospitals in Jiangsu Province and China.

Both *P. ovale* and *P. malariae* species were reported across the south, central and north parts of Jiangsu Province. In Changzhou, Lianyungang, Yangzhou and Nantong prefectures, which accounted for more than half of all reported malaria cases, the prefecture CDCs should strengthen management of personnel at entry-exit inspection and quarantine department to obtain more information about migrant workers returning from high malaria-endemic areas such as sub-Saharan Africa and Southeast Asia. These cities have many construction enterprises and local migrant labourers frequently travel to Africa for work, and return to China infected with malaria [32, 33]. At the provincial level, since 2012, the JIPD has established a dynamic information platform for local CDCs to collect and input information on migrant workers returning from abroad. Through the platform, timely and accurate information on migrant workers is collected and analysed. Furthermore, local CDCs should also strengthen early malaria detection, standardized treatment and follow-up of those individuals who have travelled with confirmed imported malaria cases. Local hospitals should improve clinical diagnosis and treatment of imported malaria and reduce incidence of severe cases and death cases.

The geographical distribution of imported *P. ovale* sp. and *P. malariae* within Africa were mainly concentrated in western Africa. The top three countries that contributed most numbers of *P. ovale* sp. and *P. malariae* cases were Equatorial Guinea, Nigeria, and Angola. Given the large economic investment from Jiangsu to Africa, exported migrant workers for construction and labour is increasing as more Chinese companies support infrastructure projects and development [34]. Unexpectedly high sero prevalence of *P. ovale* and *P. malariae* was found in healthy West African populations [26]. Isolation and characterization of the *msp1* genes from *P. ovale* and *P. malariae* has also been reported in a significant number of blood donors in Cameron [35]. That suggested why a greater proportion of imported malaria cases originate from western Africa. In Southeast Asia, this study found Pakistan and Brunei where *P. ovale* sp. still exist and *P. malariae* was only found from Pakistan.

Sutherland considered that the weight of evidence favors the proposition that the two *P. ovale* sp. types are actually two distinct species [36]. For *P. ovale curtisi*, this study found that Nigeria was the most commonly identified place of origin, which is consistent with observational study [37]. For *P. ovale wallikeri*, imported infections were mainly clustered in Angola. This is largely because Nigeria and Angola are major economic investment locations for China and Chinese migrant labourers [38]. In Nolder et al., *P. ovale* sp. interspecies differences in the latency period were significant [37]. However, this study did not find a statistically significant difference in the latency period analysis between *P. ovale curtisi* and *P. ovale wallikeri* infections. One possible reason is that this study used patients’ symptoms onset time to calculate latency period instead of diagnostic time, which was introduced by Nolder et al. [37].

## Conclusions

This study indicates that the number of malariae and ovale malaria cases increased yearly, as well as the proportion of cases of *P. malariae* and *P. ovale* sp. out of the total. Although the majority of imported malaria cases were due to *P. falciparum*, these findings should raise awareness for all levels of CDCs to focus on accurate and timely diagnosis and surveillance of *P. ovale* and *P. malariae* infections originating from sub-Saharan Africa and Southeast Asia. The climate and environment remains suitable for the breeding of *Anopheles* in China, therefore, the threat of malaria re-introduction remains.

## Abbreviations

CCDC: China Centres for Disease Control and Prevention
CRDSIS: China routine diseases surveillance information system
IRS: indoor residual spraying
JIPD: Jiangsu Institute of Parasitic Diseases
LLIN: long-lasting insecticide nets
MDA: mass drug administration
NMEAP: National Malaria Elimination Action Plan
